# Pyrosequencing the *Bemisia tabaci* Transcriptome Reveals a Highly Diverse Bacterial Community and a Robust System for Insecticide Resistance

**DOI:** 10.1371/journal.pone.0035181

**Published:** 2012-04-30

**Authors:** Wen Xie, Qing-shu Meng, Qing-jun Wu, Shao-li Wang, Xin Yang, Ni-na Yang, Ru-mei Li, Xiao-guo Jiao, Hui-peng Pan, Bai-ming Liu, Qi Su, Bao-yun Xu, Song-nian Hu, Xu-guo Zhou, You-jun Zhang

**Affiliations:** 1 Department of Plant Protection, Institute of Vegetables and Flowers, Chinese Academy of Agricultural Sciences, Beijing, People’s Republic of China; 2 Key Laboratory of Genome Sciences and Information, Beijing Institute of Genomics, Chinese Academy of Science, Beijing, People’s Republic of China; 3 Department of Entomology, University of Kentucky, Lexington, Kentucky, United States of America; University of Crete, Greece

## Abstract

**Background:**

*Bemisia tabaci* (Gennadius) is a phloem-feeding insect poised to become one of the major insect pests in open field and greenhouse production systems throughout the world. The high level of resistance to insecticides is a main factor that hinders continued use of insecticides for suppression of *B. tabaci*. Despite its prevalence, little is known about *B. tabaci* at the genome level. To fill this gap, an invasive *B. tabaci* B biotype was subjected to pyrosequencing-based transcriptome analysis to identify genes and gene networks putatively involved in various physiological and toxicological processes.

**Methodology and Principal Findings:**

Using Roche 454 pyrosequencing, 857,205 reads containing approximately 340 megabases were obtained from the *B. tabaci* transcriptome. *De novo* assembly generated 178,669 unigenes including 30,980 from insects, 17,881 from bacteria, and 129,808 from the nohit. A total of 50,835 (28.45%) unigenes showed similarity to the non-redundant database in GenBank with a cut-off E-value of 10–5. Among them, 40,611 unigenes were assigned to one or more GO terms and 6,917 unigenes were assigned to 288 known pathways. *De novo* metatranscriptome analysis revealed highly diverse bacterial symbionts in *B. tabaci*, and demonstrated the host-symbiont cooperation in amino acid production. In-depth transcriptome analysis indentified putative molecular markers, and genes potentially involved in insecticide resistance and nutrient digestion. The utility of this transcriptome was validated by a thiamethoxam resistance study, in which annotated cytochrome P450 genes were significantly overexpressed in the resistant *B. tabaci* in comparison to its susceptible counterparts.

**Conclusions:**

This transcriptome/metatranscriptome analysis sheds light on the molecular understanding of symbiosis and insecticide resistance in an agriculturally important phloem-feeding insect pest, and lays the foundation for future functional genomics research of the *B. tabaci* complex. Moreover, current pyrosequencing effort greatly enriched the existing whitefly EST database, and makes RNAseq a viable option for future genomic analysis.

## Introduction

The whitefly, *Bemisia tabaci* (Gennadius) (Hemiptera: Aleyrodidae), is a phloem-feeding insect pest that causes severe damage in both agricultural and horticultural systems worldwide. More than 24 morphologically indistinguishable *B. tabaci* biotypes have been identified [1], and recent studies suggest that most of these biotypes represent genetically distinct cryptic species [2]–[5]. Among them, B biotype (also known as Middle East-Asia Minor 1) [2] has been studied extensively and considered as one of the most invasive and destructive whiteflies worldwide. As an invasive species, *B. tabaci* can cause considerable yield losses dierctly through phloem-feeding and indirectly through the transmission of plant pathogenic begomoviruses [6]. *Bemisia tabaci* has been controlled predominantly by synthetic insecticides. Due to consistent exposure to insecticides, *B. tabaci* has developed high levels of resistance to a wide range of commonly used synthetic insecticides [7]–[11]. In China, *B. tabaci* was first recorded in the late 1940s, and the indigenous whiteflies have never elevated to the major pest status. However, this has changed when *B. tabaci* B biotype, originated in the Middle East-Asia Minor region including Iran, Israel, Jordan, Kuwait, Pakistan, Saudi Arabia, Syria, United Arab Republic, and Yemen, was introduced into the mainland China in the mid-1990s. Since then, B biotype has gradually displaced the indigenous whiteflies in most parts of the China [12], [13], and has become a major insect pests in the open field as well as in the greenhouse production systems.

Same as phloem-feeding aphids, *B. tabaci* not only harbors *Portiera aleyrodidarum*, an obligatory symbiotic bacterium which supplements the amino acid deficient diets [14], but also has a diverse array of facultative symbionts including *Rickettsia, Hamiltonella, Wolbachia, Arsenophonus, Cardinium* and *Fritschea*. Although these facultative symbionts are considered “secondary” because they are not essential for the survival of their hosts, they can manipulate hosts in many other ways, and bear evolutionary significance [15]. Chiel *et al* surveyed the composition of facultative symbionts among laboratory and field populations of *B. tabaci* from various host plants in Israel [16]. The results established a correlation between facultative symbionts and whitefly biotypes, e.g., *Hamiltonella* was B -specific, whereas *Wolbachia* and *Arsenophonus* were detected only in the Q biotype. Such association suggests a possible contribution of these facultative bacterial symbionts to the biological differences observed among whitefly biotypes, including insecticide resistance, host range, competitive displacement, virus transmission, and speciation [16]. Morin *et al* showed that a 63-kDa GroEL homologue produced by the endosymbiotic *Hamiltonella* is essential for the circulative transmission of the *Tomato yellow leaf curl virus* (TYLCV) in *B. tabaci* [17]. Interactions between GroEL protein and TYLCV particles ensure the safe circulation of the virus in insect hemolymph [17]–[19]. In Israel, *Hamiltonella* has only been detected in B biotype [16], and B can efficiently transmit the virus. In contrast, Q does not harbor *Hamiltonella*, and it can barely transmit TYLCV. Based on these observations, a causal link between the transmission efficiency of TYLCV and the presence of *Hamiltonella* has been established [18]. In southwestern United States, the range expansion of *B. tabaci* B biotype is apparently facilitated by the rapid spread of *Rickettsia* sp. nr. *Bellii*, a maternally inherited facultative bacterial symbiont [15]. Whiteflies infected with *Rickettsia* had a significantly higher fitness level and *Rickettsia* can manipulate the sex ratio of whitefly hosts by producing female-biased offspring. This dynamic interactions between *Rickettsia* and their whitefly hosts represent a rapid coevolution of both insects and their symbionts to optimize the newly established symbiosis [15].

Despite its ever-increasing pest status and enormous economic impacts, the whole genome sequencing of *B. tabaci* has yet to be materialized as a result of its innate complexity in biology (symbiosis) and genome (genome size, repeat elements, and high levels of heterozygosity). Next-generation sequencing (NGS) [20]–[22], including Roche 454-based pyrosequencing and Solexa/Illumina-based deep sequencing, have provided unprecedented opportunities for genomic research in non-model systems wherein little or no genomic resources are available [23]. For example, NGS has been applied in various transcriptomic analyses in insects and has contributed substantially to gene discovery, including molecular markers (SNPs) [24], *Bt* receptors [25], rice stripe virus identification [26], cyanogenic glucosides biosynthesis [27], immune responses [28], [29], chemosensation and sex determination [30], insecticide targets and detoxifying enzymes [31]–[33], developmental stage -specific genes [34], [35] and tissue-specific genes [36], [37] (Table S1). Transcriptomic analysis of the greenhouse whitefly, *Trialeurodes vaporariorum*, uncovered a diverse array of transcripts potentially involved in the xenobiotics detoxification and the targets for major classes of synthetic insecticides [31]. Most recently, the in-depth analyses of *B. tabaci* transcriptomes gave a better understanding of molecular mechanisms underlying the conspecific divergence of the B and Q biotypes [32], [38].

In this study, we used the Roche 454 pyrosequencing platform to provide a comprehensive view of the genes expressed in an invasive *B.tabaci* B biotype. We generated over 300 million bases of high-quality DNA sequences and carried out transcriptome and metatranscriptome analyses to shed light on the molecular bases of symbiosis and insecticide resistance. Moreover, this transcriptome sequencing effort has significantly enriched the existing gene pool for this agriculturally important key pest and provides an invaluable resource for the subsequent RNAseq analysis as well as for the future *B. tabaci* genome annotation.

## Results and Discussion

### Sequencing Summary

To obtain a comprehensive view of the transcriptional profile of the invasive sweet potato whitefly, *Bemisia tabaci,* B biotype in China, a polyphenic cDNA library including egg, nymph, and adult developmental stages was constructed and sequenced using the Roche 454 GS FLX Titanium platform. One picotiter plate of sequencing generated 1,109,732 raw reads with an average sequence length of 304 bases and an average GC content of 39%. After trimming the low quality sequences and removing the rRNAs and short reads of less than 100 bps, 907,985 reads were passed through to the next process. In addition, 50,780 reads mapped to *B. tabaci* mitochondrion were discarded. Eventually, a total of 857,205 reads were generated and used for the subsequent assembly.

A “step-by-step” strategy was used in the assembly of the *B. tabaci* transcriptome to accurately distinguish the sequences from different origins including insect, gut symbionts, parasites, and pathogens. After the assembly, 178,669 unigenes were obtained including 23,694 isotigs and 154,975 singletons (Table 1; Figure S1). Among them, 30,980 unigenes (6781 isotigs and 24199 singletons) belonged to the insect group. The length of isotigs varied from 180 to 4,681 bp with an average length of 840 bp. The sequencing coverage (estimated as the average number of reads per isotig) was 80 for the insect group. For the bacteria and nohit groups, we obtained 523 and 16390 isotigs, respectively, and 17,358 and 113,418 singletons, respectively. The sequencing coverage for the bacteria and nohit group was 17 and 43, respectively. The average lengths of the isotigs for the insect and nohit groups are comparable to the average length of the *B. tabaci* contigs documented in the traditional Sanger sequencing method [39].

**Table 1:** Sequence and assembly summary of *B.tabaci* transcriptome.

Sequence and assembly summary	Insect group	Bacteria group	No-hit group	Total
Total reads	317497	26003	513705	857205
Aligned reads	289514	8407	389169	687090
Number of isotigs	6781	523	16390	23694
Average isotig length (bp)	840	595	732	/
Range of isotig length (bp)	180–4681	135–4004	101–5775	/
Average number of reads per isotig	80	17	43	/
Range of number of reads per isotig	2–3669	2–844	2–3013	/
Number of singletons	24199	17358	113418	154975
Number of unigenes (contigs+singletons)	30980	17881	129808	178669
Unigenes with NCBI nr match^1^	29672	17607	3556	50835

1 The cutoff value was 1e-5.

### Functional Annotation

**Figure 1:**
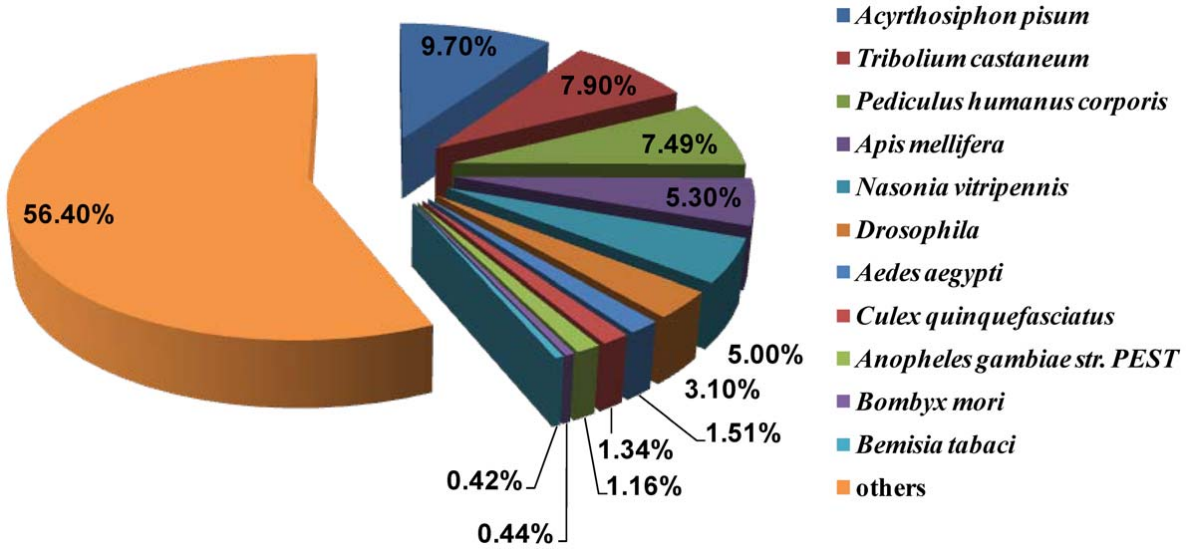
Top BLAST hits of *B. tabaci* sequences to various insect species.

The unigenes were subjected to BLASTX similarity search against the NCBI non-redundant (nr) protein database to determine their putative functions. Based on the sequence similarity with known insect and bacteria genes, unigenes were subdivided into three groups: insect (30,980), bacteria (17,881), and nohit (129808). A total of 50,835 unigenes (28.5%) in these three groups exhibited significant similarity at the cutoff value of 1e-5 (Table 1, Table S2). The taxonomic distribution of species that provided the most top hits is shown in Figure 1, and the species that generated most of the top BLAST hits was the pea aphid (*Acyrthosiphon pisum*) (9.70%) [40]. A high degree of sequence similarity between *B. tabaci* and *A. pisum* may be due to their similarity in the taxonomic status (Hemiptera), diet/feeding behavior (phloem-feeding), and symbiotic relationships with their gut microbiota (obligatory and facultative symbiosis).

### Functional Classification and Pathway Analysis

**Figure 2:**
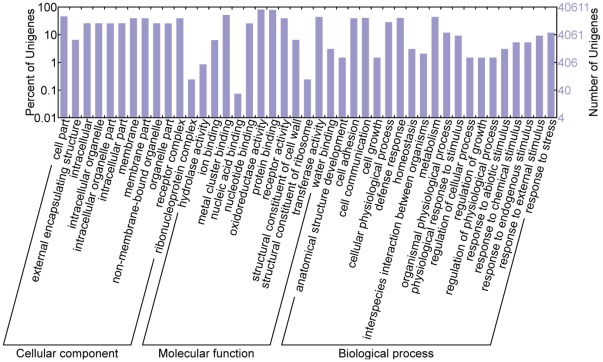
Distribution of unigenes based on the Gene Ontology (GO) functional categories.

In total, 40,611 unigenes were assigned to one or more Gene Ontology (GO) terms (Table S3). As shown in Figure 2, these unigenes were divided into three main categories: cellular component (12 subcategories), molecular function (12 subcategories), and biological process (19 subcategories). The largest subcategory found in the “cellular component” group was “cell part,” which comprised 45.9% of the genes. This is consistent with the bed bug, *Cimex lectularius* [33], emerald ash borer, *Agrilus planipennis* [36], and Q-biotype whitefly [32]. In the “molecular function” and “biological process” GO terms, “oxidoreductase activity (81.9%)” and “metabolism (44.0%)” were the most abundant subcategory, respectively. The Kyoto Encyclopedia of Genes and Genomes (KEGG) metabolic pathway analysis revealed that 6,917 unigenes could be assigned to the 288 given pathways (Table S4). The metabolic pathways putatively involved in insecticide resistance and nutrient digestion were metabolism of xenobiotics, drug metabolism, salivary secretion, peroxisome, and ABC transporters.

### Putative Molecular Marker

In total, 9,075 simple sequence repeats (SSRs or microsatellites) including 1470 (16%) trinucleotide repeats, 671 (7.4%) dinucleotide, and 196 (2.2%) tetranucleotide repeats were identified (Table 2; Table S5). In addition, 7,834 unigenes contained SSRs, in which 902 (12%) had more than 1 SSR. The molecular marker, SSRs, identified in this study lays a foundation for the better understanding of the adaptation and ecology of *B. tabaci* [41]. The identity of predicted molecular markers, however, needs to be validated in future research to exclude false positives and sequencing errors.

**Table 2:** Summary of microsatellite loci predicted in *B.tabaci* transcriptome.

Total number of sequences examined	178669
Total size of examined sequences (bp)	64757967
Total number of identified SSRs	9075
Number of sequences containing SSR	7834
Number of sequences containing more than 1 SSR	902
Number of SSRs present in compound formation	1052

### Meta-transcriptome Analysis of Symbiotic Bacteria

**Figure 3:**
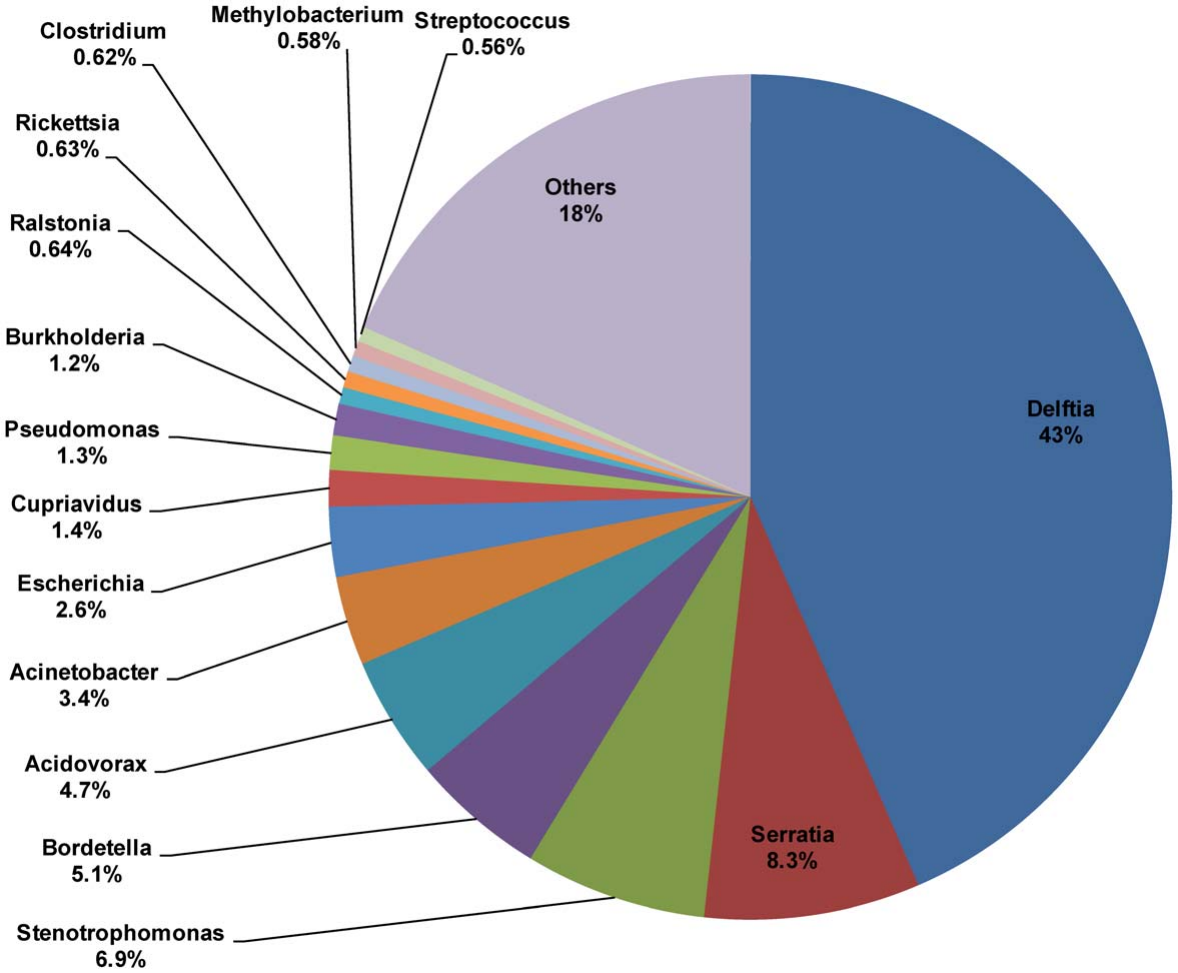
Diversity and phylogeny of bacterial symbionts in *B. tabaci*. Phylogenetic relationships of symbiotic bacteria in *B.tabaci* were resolved at the Genus levels.

A total of 17,766 bacterial unigenes were classified into 322 genera, suggesting a rich microbial community in *B. tabaci*. The most abundant phylum, class, order, family, and genus was Proteobacteria (92%) (Figure S2), Betaproteobacteria (59%) (Figure S3), Burkholderiales (58%) (Figure S4), Comamonadaceae (50%) (Figure S5), and Delftia (43%) (Figure 3, Table S6), respectively. *Delftia sp.*, a gram negative bacterium which belongs to the Proteobacteria, was detected only in insect’s hemolymph [42]. *Delftia* sp. is a known D-amino acid amidase-producing bacterium and might play a key role in insect survival [43], [44]. *Delftia, Wolbachia,* and three other cultivable bacteria co-occurred and persisted in the guts of *Aedes albopictus* [45]. *Delftia* was also documented in the gut microfauna of *Helicoverpa armigera* [46] and in the metagenome of *Daphnia* symbionts [47]. Other abundant genera included *Serratia*, *Stenotrophomonas*, and *Bordetella*. *Delftia* and *Serratia* were all detectable in *H. vitripennis* [48]. *Serratia* contains a secondary endosymbiont, *Serratia symbiotica*. *Serratia symbiotica* provided protection against heat stress in several aphid species [49], [50], and might be related to the biosynthesis of tryptophan in *Cinara cedri* [51]. *Stenotrophomonas* is a midgut bacterium in *Anopheles gambiae* [52] and *Culex quinquefasciatus* [53]. In comparison to prothiofos-resistant populations, susceptible *Plutella xylostella* lacked *Pseudomonas sp.* or *Stenotrophomonas sp*. in their gut microbiota, bacteria species known for their ability to break down pesticides [54]–[56]. Same as *Delftia, Bordetella* was documented in the metagenome of *Daphnia* symbionts as well [47]. Endosymbionts, including *Hamiltonella, Buchnera,* and *Spirosoma*, were prevalent in phloem-feeding aphid [57]–[60] and *B. tabaci* (Table 3 and Table S6).

**Table 3:** Genes of interest in *B.tabaci* transcriptome.

Candidate genes	Unigenes (isotigs)^1^	Unique hits	Average lengthof unigenes (bp)	Average lengthof isotigs (bp)
*Metabolic resistance and insecticide targets*				
Cytochrome P450 monooxygenase	223 (35)	97	497	1146
Carboxylesterase	45 (18)	17	719	1274
Glutathione S-transferase	60 (20)	24	564	914
Acetylcholinesterase	7 (1)	4	338	642
Nicotinic acetylcholine receptor	12 (3)	8	498	803
GABA receptor	4 (2)	3	613	719
Sodium channel	1 (1)	1	1039	1039
Chloride channel	17 (2)	14	441	703
NADH dehydrogenase	138 (59)	72	495	706
NADH oxidoreductase	10 (0)	6	371	0
ABC transporter	460 (16)	221	365	626
Catalase	21 (7)	18	560	1007
Peroxidase	48 (12)	28	416	622
Superoxide dismutase	31 (3)	15	377	744
*Digestive enzymes*				
Trypsin	16 (2)	9	432	864
Protease	329 (69)	182	470	887
Salivary protein	12 (5)	4	544	691
Sucrase	45 (13)	8	522	864
Trehalase	48 (4)	14	386	797
*Symbiotic bacteria*				
*Wolbachia*	34 (4)	12	398	660
*Rickettsia*	137 (3)	77	330	740
*Portiera*	96 (35)	31	450	820
*Hamiltonella*	112 (1)	58	322	620
*Arsenophonus*	9 (0)	5	275	0

1 Unigenes include both isotigs and singletons. Here, we summarized the total number of unigenes (isotigs), total number of unique BLAST hits in the NCBI nr database with a cutoff value of 1e-5, average length of unigenes, and average length of isotigs within unigenes.

Table 3 summarized partial sequences of some bacterial endosymbionts in *B. tabaci*, including *Portiera* (96 unigenes), *Wolbachia* (34 unigenes), *Rickettsia* (137 unigenes), *Hamiltonella* (112 unigenes) and *Arsenophonus* (9 unigenes). To date, one primary symbiont (*Portiera*) and six secondary symbionts (*Hamiltonella*, *Arsenophonus*, *Cardinium*, *Wolbachia*, *Rickettsia*, and *Fritschea*) have been documented in *B. tabaci* [61]. The primary symbiont could supplement nutrients when the host insects fail to provide sufficient quantity of nutrients from a restricted diet of plant phloem [61], [62]. These secondary symbionts play important roles in the biology and ecology of *B*. *tabaci* as well. For example, single or repetitive infections with secondary symbionts can affect the susceptibility of *B. tabaci* to synthetic insecticides [63], [64]. The proliferation of *Rickettsia* may be involved in *B. tabaci*’s ability to defend against natural enemies [65] and influence the thermotolerance in B biotype [66]. *Hamiltonella* was reported to provide the protection for *A. pisum* against parasitoids [67]–[69], and was involved in the transmission of the *tomato yellow leaf virus* by *B. tabaci* B biotype [18]. It is worth noting that 9 *Arsenophonus* sequences were found in this study, while a previous study showed that *Arsenophonus* was Q biotype-specific in Israel [16]. However, this is not unusual that the composition of facultative symbionts in whitefly is biotype dependent and geographically dependent. For example, *Hamiltonella* was B-specific in Israeli populations [16], whereas it was ubiquitously abundant in both B and Q biotypes in China [70].

### Host-symbiont cooperation in the amino acid production

**Figure 4:**
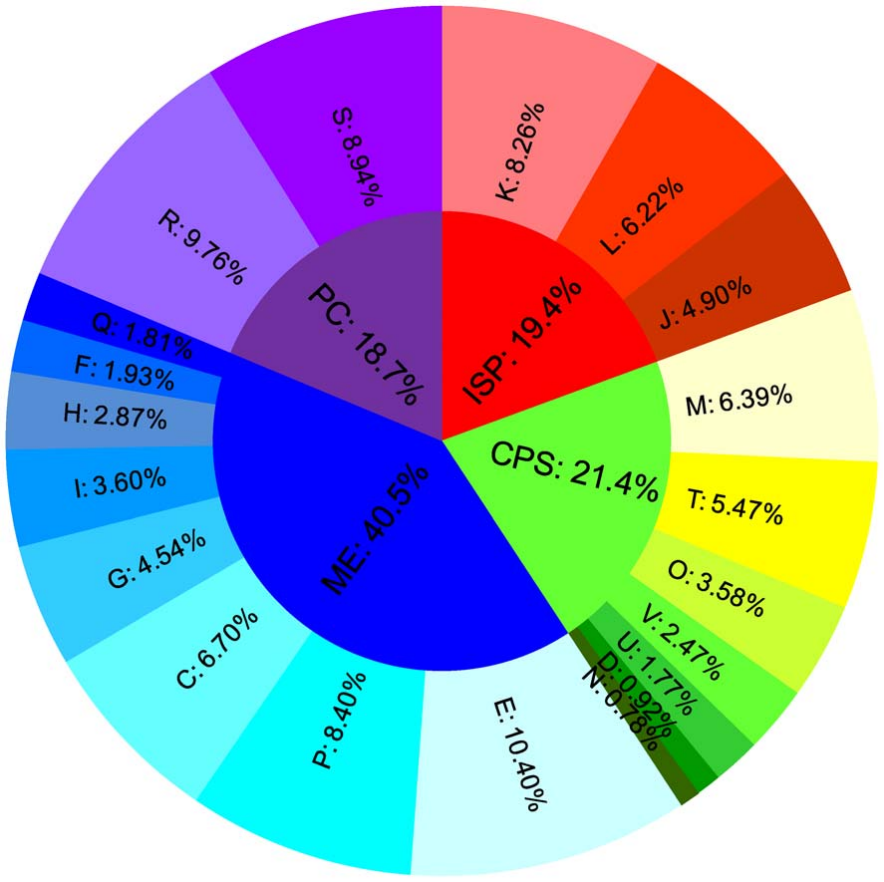
COG classification of the symbiotic bacteria in *B. tabaci*. ME: Metabolism; CPS: Cellular processes and signaling; ISP: Information storage and processing; PC: Poorly characterized (inside circle); Q: Secondary metabolites biosynthesis, transport and catabolism; F: Nucleotide transport and metabolism; H: Coenzyme transport and metabolism; I: Lipid transport and metabolism; G: Carbohydrate transport and metabolism; C: Energy production and conversion; P: Inorganic ion transport and metabolism;E: Amino acid transport and metabolism; N: Cell motility; D: Cell cycle control, cell division, chromosome partitioning; U: Intracellular trafficking, secretion, and vesicular transport; V: Defense mechanisms; O: Posttranslational modification, protein turnover, chaperones; T: Signal transduction mechanisms; M: Cell wall/membrane/envelope biogenesis; J: Translation, ribosomal structure and biogenesis; L: Replication, recombination and repair; K: Transcription; S: Function unknown; R: General function prediction only.

For functional classification, bacterial unigenes were subjected to the Cluster of Orthologous Groups (COG) database. A total of 9,647 unigenes have a COG classification in four main categories and 20 subcategories (Figure 4). The most enriched category is “METABOLISM,” which account for 40.5% of the unigenes involved in all COG categories. The cluster “Amino acid transport and metabolism” represents the largest subcategory (10.4%). In aphids, the 10 essential amino acids are scarce in the phloem sap diet and are supplied by the obligate bacterial endosymbiont [71].

**Figure 5:**
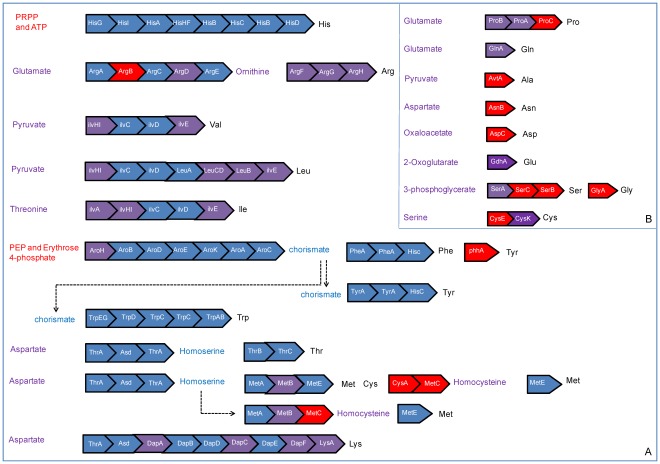
Schematic drawing of amino-acid biosynthetic pathways in *B.tabaci*. Essential (A) and non-essential (B) amino acid biosynthetic pathways were deduced from *B.tabaci* transcriptome sequences. The sequential pathways are represented by boxes which indicate one step catalyzed by the named enzyme. Blue box denotes the enzyme detected exclusively in the bacterial group, red box represents the enzyme only detected in the insect group, and purple box designates for the enzyme found in both groups. Outside the boxes, the three-letter abbreviations of amino acids are highlighted in black; compounds detected only in the insect group are in red; compounds found only in the bacterial group are in blue; and compounds detected in both groups are in purple.

We found 533 KEGG Orthology (KO) numbers involved in the amino-acid biosynthesis, among which 276 from the insect group and 257 from the bacterial group. Not surprisingly, genes for biosyntheses of the amino acids essential for the whitefly host are predominantly supplied by the bacterial group (Figure 5A) and those for the non-essential amino acids are predominantly supplied by the insect group (Figure 5B). Such complementarity signifies the beauty of symbiosis, in which symbiont and host are interconnected and interdependent in the production of essential and non-essential amino acids, respectively. In some extreme cases, the biosynthetic pathways of the host and the symbiont are intertwined, such as glutamate and aspartate (Figure 5A), a group of non-essential amino acids serving as the precursors for an array of essential amino acids. This result is in agreement with aphids [71], [72], suggesting the collaborative productions of essential and non-essential amino acids in bacterial symbionts and their insect hosts could be the rule rather than the exception in the phloem-feeding insects.

### Genes Putatively Involved in Insecticide Resistance

Invasive biotypes of *B. tabaci* have developed substantial resistance to a wide range of synthetic insecticides, especially to the neonicotinoids, over the past decade. The rapid competitive displacement of the invasive *B. tabaci* over the indigenous counterparts in China is explained, at least partially, by the onset development of insecticide resistance. Sequences encoding enzymes potentially involved in the xenobiotics detoxification and the targets of the major classes of synthetic insecticides were extracted and compared with sequences from the NCBI protein database. Genes potentially involved in the insecticide metabolic resistance are summarized in Table 3, including conventional detoxification enzymes such as cytochrome P450 monooxygenase (P450, 223 unigenes), carboxylesterase (CarE, 45 unigenes) and glutathione s-transferase (GST, 60 unigenes); and putative insecticide targets, including nicotinic acetylcholine receptor (nAChRs, 12 unigenes), gamma-aminobutyric acid receptor (GABA, 4 unigenes) and acetylcholinesterase (AchE, 7 unigenes). The average length of these unigenes was 497 (P450), 719 (CarE), 564 (GST), 498 (nAChRs), 613 (GABA), and 338bp (AchE) respectively. It is worth noting that an array of ABC transporters (460 unigenes with an average length of 365bp) was also indentified in the *B. tabaci* transcriptome, representing a gene subfamily that plays a key role in xenobiotic resistance [73].

P450s are a large superfamily of heme-containing monooxygenases that play a critical role in catalyzing the metabolisms of endogenous and exogenous compounds [74], [75]. Based on the closest BLAST hits in the NCBI nr database, transcripts encoding putative P450s were assigned to appropriate CYP clades and families (Table 4). All 4 major insect CYP clades (CYP2, 3, 4 and mitochondrial; [76]–[78]) were identified in *B. tabaci* transcriptome. Specifically, among 223 P450 unigenes annotated in the NCBI nr database, 135 contained CYP family information, of which 4 unigenes were excluded from the subsequent analysis due to their sequence similarity with non-insect organisms including *Arabidopsis thaliana* and *Uncinocarpus reesii* 1704. The remaining 131 unigenes were subdivided into 4 clades and 13 families, including 4 families of CYP18, CYP304, CYP305 and CYP306 in CYP2 clade, 3 families of CYP6, CYP347 and CYP354 in CYP3 clade, 1 CYP4 family in CYP4 clade, and 5 families of CYP49, CYP301, CYP302, CYP315 and CYP353 in mitochondrial CYP clade (Table 4). The majority of annotated P450s belonged to the CYP3 clade (84/131), and followed by CYP4 (26/131), mitochondrial (15/131), and CPY 2 (6/131). It is not surprise to see the dominance of CYP3 and CYP4 P450 clades due to their documented functions in the metabolism of plant secondary chemicals and synthetic insecticides [76]. At family level, CYP6 (78) and CYP4 (26) are the most abundant P450 families. Recent mechanistic study showed that the neonicotinoid resistance developed in phleom-feeding hemipterans *B. tabaci* and *Myzus persicae* were caused by the overexpression of CYP6CM1 [89] and CYP6CY3 [79], respectively. In comparison to other phloem-feeding hemipterans (115 in the green peach aphid *M. persicae*, 83 in the pea aphid *Acyrthosiphon pisum*), the number of putative P450s annotated in the *B. tabaci* transcriptome (131) is well within the range [80]. Without a fully sequenced genome, however, this number is likely overestimated and the identity of these annotated P450s warrant further analysis and validation. Most recently, 454-based transcriptomic analysis of greenhouse whitefly *T. vaporariorum* identified 57 P450s, although authors suspected the number of P450s should be greater [31].

**Table 4:** Putative P450s in *B. tabaci* transcriptome.

Putative P450s		# Occurrence	Family members with corresponding numbers	
Clade	Family			
CYP2	CYP18	3	CYPXVIIIA1(3)	
	CYP304	1	CYPCCCIVA1(1)	
	CYP305	1	CYPCCCVA1(1)	
	CYP306	1	CYPCCCVIA1(1)	
CYP3	CYP6	78	CYPVIX1(2), CYPVI-like(12), CYPVIG2(2), CYPVICX1(16), CYPVICM1(21), CYPVIBQ13(3), CYPVIBK17(5), CYPVIAY1(5), CYPVIAX1(7), CYPVIAS33(2), CYPVIA8(1), CYPVIA5(2)	
	CYP347	5	CYPCCCXLVIIA1(5)	
	CYP354	1	CYPCCCLIVA5(1)	
CYP4	CYP4	26	CYPIVV2(1), CYPIV-like(12), CYPIVL6(1), CYPIVG48(1),CYPIVG43(1),CYPIVG11(1), CYPIVC39(1), CYPIVC3(1), CYPIVC1(6), CYPIV(1)	
Mitochondrial CYP	CYP49	2	CYPXLIXA1(2)	
	CYP301	1	CYPCCCIB1(1)	
	CYP302	9	CYPCCCIIA1(9)	
	CYP315	2	CYPCCCXVA1(2)	
	CYP353	1	CYPCCCLIIIA1(1)	

### A Case Study with Thiamethoxam Resistant *B. tabaci*

Neonicotinoids, targeting the postsynaptic nicotinic acetylcholine receptors (nAChRs), are one of the most effective insecticides against a broad spectrum of phloem-feeding insects, including hemipterans like aphids and whiteflies [81]. Thiamethoxam was the first commercially available neonicotinoid insecticide from the thianicotinyl subclass [82]. Several whitefly species, including *B. tabaci* and *Trialeurodes vaporariorum*, have developed substantial resistance to neonicotinoids [11], [83], [84]. Alteration of target sites and metabolic resistance are the two major mechanisms governing insecticide resistance [85]. In the case of neonicotinoid resistance, both mutations in nAChRs [86] and elevated metabolic detoxification have been found to be involved [87], [88]. The mechanistic study of neonicotinoid resistance in *B. tabaci*, however, only linked the enhanced oxidative detoxification by P450 rather than the target site mutation to the resistance [89], [90].

**Figure 6:**
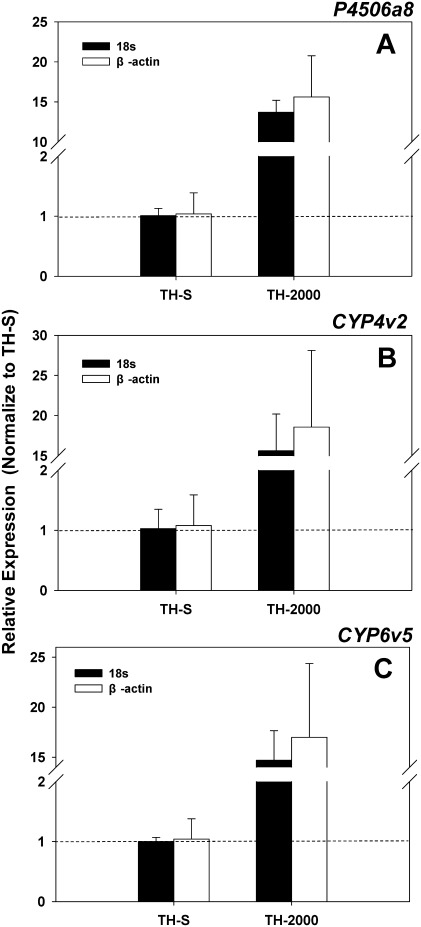
Gene expression profiling of putative P450s in thiamethoxam resistant and susceptible *B. tabaci*. mRNA expression levels of *P4506a8* (A), *CYP4v2* (B), and *CYP6v5* (C) in thiamethoxam resistant (TH-2000) and susceptible **(**TH-S) *B. tabaci* were analyzed by the qRT-PCR. Relative gene expressions in the resistant TH-2000 population were normalized to the susceptible TH-S population using both *18s rRNA* and *β-actin* as the internal references. Standard errors were generated from the three biological replicates.

To investigate the expression profiles of putative detoxification P450s in the thiamethoxam-resistant [TH-2000, LC_50_ (95% CL) = 1326 (1259–1615) mg L^–1^] and susceptible strains [TH-S, LC_50_ (95% CL) = 17.2 (15.4–19.3) mg L^–1^], 44 P450 genes from this study, several others from the traditional Sanger sequencing (4) [39], and NCBI database (15) were subjected to the qRT-PCR analyses (Table S7). Three P450 genes, *CYP6a8*, *CYP4v2* and *CYP6v5*, showed significantly higher mRNA expression levels (>10-fold) in the resistant TH-2000 in comparison to the susceptible TH-S strain (Table S7; Figure 6). *CYP6cm1*, which was over-expressed in the imidacloprid-resistant *B. tabaci* [89], did not exhibit elevated mRNA levels (Table S7). This may suggest a different mechanism existed between the thiamethoxam and imidacloprid resistance, although both insecticides are neonicotinoids. To elucidate the role of these P450s in the *B. tabaci* thiamethoxam resistance, studies including cloning the full length cDNA and functional characterization of *CYP6a8*, *CYP4v2*, and *CYP6v5* using RNAi are currently in progress.

### Putative Digestive Enzymes

Furthermore, *B. tabaci* has more than 600 recognized host plants [91], suggesting a diverse array of digestive enzymes. Digestive enzymes identified from this sequencing effort include trypsin (16 unigenes), protease (329 unigenes), salivary protein (12 unigenes), sucrase (45 unigenes), and trehalase (48 unigenes) (Table 3). For example, trehalase plays a pivotal role in various physiological processes, including flight metabolism [92], chitin synthesis [93], and cold tolerance [94] through the hydrolysis of trehalose, a principal hemolymph sugar in insects which is an indispensable substrate for energy production and macromolecular biosynthesis [95]. Trehalase was divided into the soluble (Tre-1) and the membrane-bound (Tre-2) trehalases [96]. In this study, the 48 unigenes were manually assembled into 14 different trehalases containing both Tre-1 and Tre-2 (Table S8).

### Transcriptome Resources in Whitefly

Current transcriptome data come from the traditional Sanger sequencing and, most recently, next generation sequencing including Roche 454 pyrosequencing and Illumina deep sequencing (Table 5). The average lengths of reads and assembled sequences (contigs/unique sequences/isotigs) are comparable in the Sanger (301 and 515 bp, respectively) and 454-based transcriptomes (362 and 965, respectively, in *T. vaporariorum*, and 304 and 760, respectively, in this study), and considerably longer than the Illumina-based sequences (75 and 479, respectively, in *B. tabaci* B-biotype, and 75 and 266, respectively, *B. tabaci* B-biotype). However, the ultra high-throughput Illumina-based transcriptomes provide exponentially more sequencing information, i.e., billion in comparison to million base pair of total reads. Despite the relatively smaller sequencing outputs, the 454 pyrosequencing performs equally well or better at addressing specific biological questions. For instance, a survey of genes putatively involved in insecticide resistance deduced from current available whitefly transcriptomes showed the similar or greater number of transcripts predicted by the 454 sequencing platform in comparison to others (Table S9).

**Table 5:** Current available whitefly transcriptomes.

Sequencing Summary	*B. tabaci*	*B. tabaci*	*B. tabaci*	*T. vaporariorum*	*B. tabaci*
Biotype	B	B	Q	N/A	B
Sequencing platform	Sanger	Illumina	Illumina	454	454
Total base pairs (bp)	2,742,110	1,278,712,500	3,279,855,000	52,832,938	337,358,528
Total number of reads	9,110	17,049,500	43,731,400	1,104,651	1,109,732
Ave. read length (bp)	301	75	75	362	304
Total number of CUI^1^	4,859	57,741	168,900	54,748	23,694
Ave. length of CUI (bp)	515	479	266	965	760
No. of Blast hits (%)^2^	4,063(83.6)	44,689 (77.4)	82,276 (48.7)	N/A	23,694 (100)
Reference	[39]	[38]	[32]	[31]	this study

1 CUI stands for Contigs/Unique sequences/Isotigs.

2 454 data from this study was Blastn with other whitefly transcriptomes using a cutoff value of 1e-5. The total number and the percentage (in parenthesis) of significant blast hits with the reference *B. tabaci* transcriptome are listed here. N/A: not available.

Unlike the whole genome, a transcriptome represents the snap shot of a physical (e.g., tissue) and/or a physiological (e.g., developmental stage) state of the tested organisms. The dynamic nature of transcriptome sequencing offers an unparallel opportunity to investigate fundamental biological questions at the global gene expression level. The transcriptome analyses of whiteflies shed light on the molecular understanding of insecticide resistance ([31] this study), conspecific divergence [32], [38], and symbiosis (this study). Without a fully sequenced genome, a robust EST database is essential for any “omics”-based analyses in whiteflies. Tissue- and treatment-specific transcriptomes will add additional dimensions to existing whitefly EST database to make RNAseq, also called “Whole Transcriptome Shotgun Sequencing”, a viable option in future analyses. Moreover, a comprehensively annotated EST library will provide molecular clues to address outstanding biological questions related to whiteflies, symbionts, and their interactions with host plants.

## Materials and Methods

### Ethics Statement

*Bemisia tabaci* B biotype strains used in this study were initially collected in Beijing in 2000, and have been maintained in a greenhouse at the Institute of Vegetables and Flowers, Chinese Academy of Agricultural Sciences. No specific permit was required for the described field collections, and the location is not privately-owned or protected in any way. The species in the genus of Aleyrodidae are common agricultural pests and are not included in the “List of Protected Animals in China”.

### Construction of a Comprehensive cDNA Library

#### Sample preparation

To maximize the representation of *B. tabaci* transcriptome, cultures of different *B. tabaci* B biotypes including strains developed on different host plants and strains resistant to thiamethoxam, abamectin, and bifenthrin resistant *B. tabaci* were collected. In addition, all development stages including egg, nymph and adult were pooled. The purity of each *B. tabaci* B biotype strain was examined using a mitochondrial DNA marker (COI) every 2–3 generations [97].

#### RNA isolation, library construction, and 454 sequencing

The RNA sample was extracted using the Trizol reagent (Invitrogen) according to the manufacturer’s instructions. The purity and degradation of total RNAs were checked on 1% agarose gels, respectively. About 15 µg of total RNA from each sample were pooled equally, producing about 270 µg of total RNA. The concentration of the pooled sample was adjusted to 1 µg/µl. Poly(A)-containing RNA was separated from the total RNA (1 µg/µl) using Dynabeads® mRNA purification kit (Invitrogen). About 1 µg of mRNA was converted to the first-strand cDNA using SuperScripe® II Reverse Transcriptase (Invitrogen) and random primers (Promega). The. cDNA synthesis of the second strand was performed using DNA polymerase I (Promega). with aRNA as the template. The amplified double cDNA product was purified and extracted using the Min Elute® Gel Extraction Kit (QIAGEN). The cDNAs were sheared to 500–1000 bp and directly used to construct a sequencing library. Approximately 1.5 µg of the resultant cDNA was end polished followed by ligation with adapters and finally immobilized on beads.

Single-strand DNA isolated from the beads was characterized for correct size using a. LabChip 7500. The concentration and proper ligation of the adapters were examined by. qRT-PCR. A full PicoTiter plate was sequenced following the manufacturer’s protocol using the Roche 454 GS FLX Titanium chemistry.

### Quantitative Real Time PCR (qRT-PCR) Analysis

#### Sample preparation

Thiamethoxam susceptible (TH-S) and resistant (TH-R) strains were determined as described previously [98], [99]. Before sample collection, a leaf-dip bioassay [98] was carried out to validate the resistance level, i.e., the resistance factor [LC50 (TH-R)/LC50 (TH-S)] was at least 70-fold. About 3,000 adult whiteflies from TH-R were treated with 2,000 mg/L thiamethoxam (∼LC80) to eliminate the heterozygous individuals. Then, the survivors were collected after 48 hours and designated as the TH-2000. A total of 300 TH-S and TH-2000 adults, respectively, were collected, snap frozen in liquid nitrogen, and stored at –80°C for the subsequent qRT-PCR analysis.

**qRT-PCR.** Total RNA was extracted from TH-2000 and TH-S adults, respectively, using Trizol (Invitrogen) following the manufacturer’s protocols. The total RNA obtained was resuspended in nuclease-free water and the concentration was measured using Nanodrop (Thermo Scientific Nanodrop 2000). About 0.5 µg of total RNA was used as template to synthesize the first-strand cDNA using a PrimerScript RT reagent Kit (TaKaRa) following the manufacturer’s protocols. The resultant cDNA was diluted to 0.1 µg/µl for further analysis in the qRT-PCR (ABI 7500) using an SYBR Green Realtime PCR Master Mix (TaKaRa).

qRT-PCR primers (Table S10) for the selected cytochrome P450 genes (Table S7) were designed using the Primer Express 2.0 software. The cycling parameter was 95°C for 30 s followed by 40 cycles of 95°C for 5 s and 62°C for 34 s, ending with melting curve analysis (65°C to 95°C in increments of 0.5°C every 5 s) to check for nonspecific product amplification. Relative gene expression of P450s was calculated by the 2^–ΔΔCt^ method [100] using housekeeping genes *18s rRNA* and *β-actin* as the references to eliminate sample-to-sample variations in the initial cDNA samples.

### Sequence Analysis

Raw data were cleaned and ribosomal RNA was trimmed with SeqClean [101] using all known rRNA sequences downloaded from the NCBI database. The minimum read length of 100bp was used to ensure assembly quality. The trimmed and size-selected reads were then mapped to *B. tabaci* mitochondrion [102] with gsMapper in Newbler 2.5 (Roche). After preprocessing, all clean reads were assembled using a step-by-step strategy. First, all known insect protein sequences (1,229,681 items) and bacteria protein sequences (19,178,750 items) were downloaded from the NCBI database. Then, the clean reads were compared with known insect and bacteria protein sequences using the BLASTX algorithm with a cutoff value of 1e-5. All clean reads were divided into three groups according to the BLAST results, which are designated as the insect groups, bacteria groups, and nohit groups. Finally, the three groups were assembled, respectively, with gsAssembler in Newbler 2.5 (Roche) using cDNA default parameter sets. For a better result, the isotigs and contigs were renamed, respectively, in the format of “2.0_BB_ISOTIG00001” and “2.0_BB_CONTIG00001” in which “2.0” stands for the second trial, ‘BB’ for the group (INSECT, BACTERIA or NOHIT), and “00001” for an arbitrarily assigned number. The singletons were renamed in the format of “2.0_BB_XXXXXXXXXXXXXX” in which each “X” denotes for an arbitrarily assigned letter or number. The Roche 454 reads of *B. tabaci* transcriptome have been deposited into the NCBI Sequence Read Archive under the accession number SRA036954.

### Functional Annotation and Classification

Unigenes including isotigs and singletons were searched against the NCBI-nr protein database using the BLASTX program with a cutoff value of 1e-5, and the best hits were regarded as the annotations of the unigenes. The unigenes that had no hits in the nr database were further compared with all existing *B. tabaci* nucleotide sequences (39,322 items) in NCBI using the BLASTN program with a default cutoff of 1e-10. For functional classification, unigenes were searched against the InterPro protein signature databases through InterProScan [103]. Unigenes with GO terms extracted from the InterPro output were classified into specific functional categories. In addition, unigenes were submitted to the KEGG online service (http://www.genome.jp/kegg/) for pathway analysis [104].

### SSR Molecular Marker Identification

The identification and localization of microsatellites were accomplished using a PERL5 script (named *MIcroSAtellite* MISA) [105]. The script can identify both perfect and compound microsatellites, which are interrupted by a certain number of bases.

### *De novo* Meta-transcriptome Analysis

The unigenes in the bacteria group were used for the *de novo* meta-transcriptomic analysis. First, the unigenes were phylogenetically classified by PhymmBL, a phylogenetic classification tool that combines Phymm and BLAST [106]. Classification results at the genus level were extracted by a Perl script with a confidence score >0.8. Then, the unigenes were searched against the COG database using BLASTX for functional classification with the cutoff value of 1e-5. Also, the unigenes in insect and bacteria groups were separately submitted to the KEGG online service (http://www.genome.jp/kegg/) [104] and BRENDA Enzyme Information System [107] for the pathway analysis of amino acids.

## Supporting Information

Figure S1 **Summary of sequencing assembly.** (A) Distribution of isotig lengths in the insect group; (B) distribution of the number of reads per isotig in the insect group;(C) distribution of isotig lengths in the bacterial group; (D) distribution of the number of reads per isotig in the bacterial group; (E) distribution of isotig lengths in the nohit group; (F) distribution of the number of reads per isotig in the nohit group.
(TIF)

Figure S2 **Diversity and phylogeny of bacterial symbionts in** ***B.tabaci*** **.** Phylogenetic relationships of symbiotic bacteria in B.tabaci were resolved at the Phylum levels.
(TIF)

Figure S3 **Diversity and phylogeny of bacterial symbionts in** ***B.tabaci*** **.** Phylogenetic relationships of symbiotic bacteria in B.tabaci were resolved at the Order levels.
(TIF)

Figure S4 **Diversity and phylogeny of bacterial symbionts in** ***B.tabaci*** **.** Phylogenetic relationships of symbiotic bacteria in B.tabaci were resolved at the Class levels.
(TIF)

Figure S5 **Diversity and phylogeny of bacterial symbionts in** ***B.tabaci*** **.** Phylogenetic relationships of symbiotic bacteria in B.tabaci were resolved at the Family levels.
(TIF)

Table S1 Transcriptome analyses in non-model insects using NGS platforms.
(DOCX)

Table S2 NCBI-nr best blast hits in the transcriptome of *B. tabaci* B biotype.
(XLSX)

Table S3 Gene ontology of *B. tabaci* transcriptome.
(XLSX)

Table S4 KEGG summary of *B. tabaci* transcriptome.
(XLSX)

Table S5 Putative microsatellite loci in *B. tabaci* transcriptome.
(XLSX)

Table S6 Genus-level distribution of *B. tabaci* bacterial sequences.
(XLSX)

Table S7 qRT-PCR analyses of selected *B. tabaci* P450s.
(XLSX)

Table S8 Putative trehalase receptor genes in *B. tabaci* transcriptome.
(DOCX)

Table S9 Genes putatively involved in insecticide resistance in whitefly transcriptomes.
(DOCX)

Table S10 Primers used for the qRT-PCR analyses.
(XLSX)
