# Supplementary figures and images for: Pyrosequencing the Bemisia tabaci Transcriptome Reveals a Highly Diverse Bacterial Community and a Robust System for Insecticide Resistance
Source: PLoS One. 2012 Apr 30;7(4):e35181. doi: 10.1371/journal.pone.0035181 (PMC3340392; doi:10.1371/journal.pone.0035181)

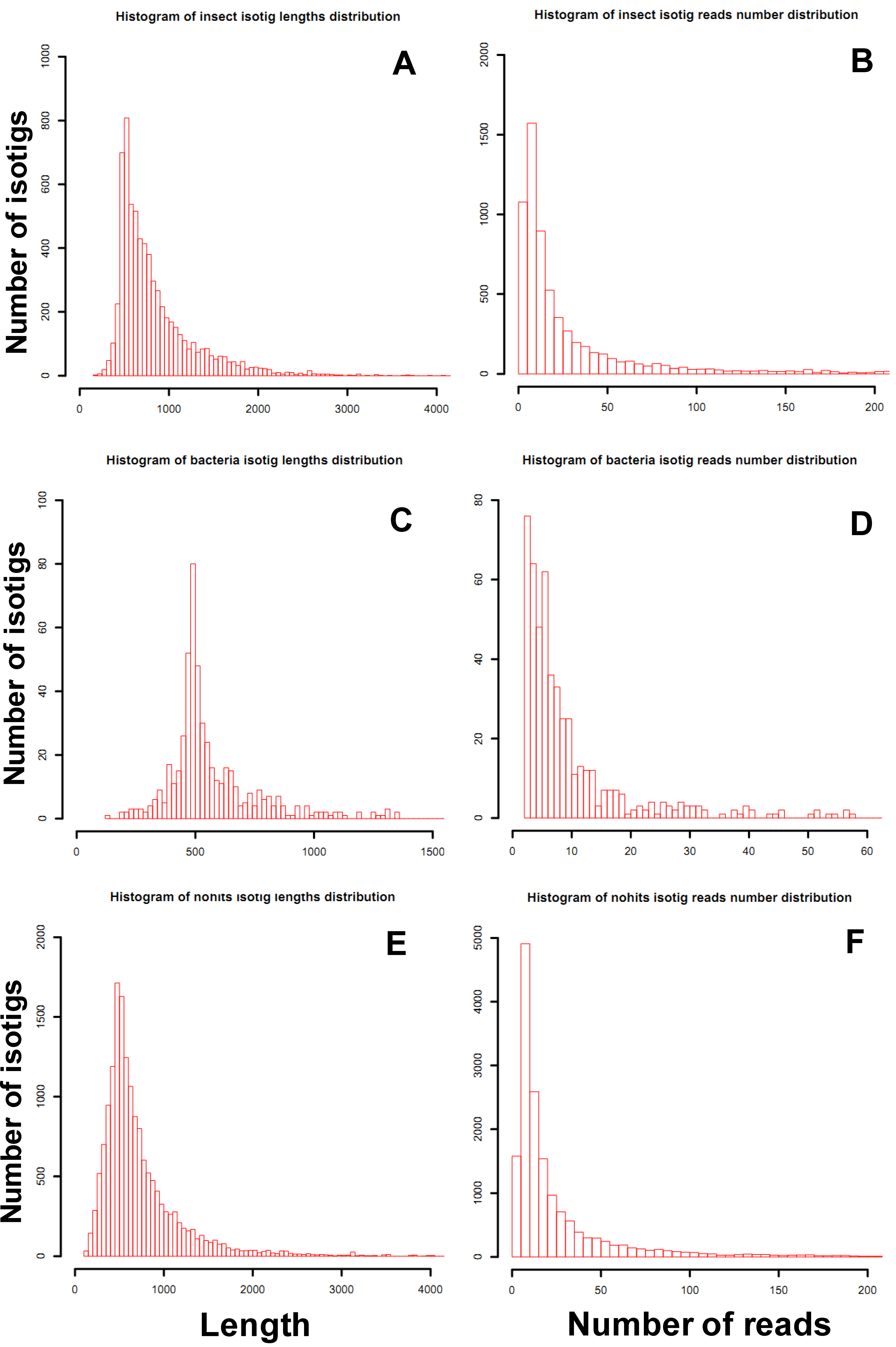

Supplement: Figure S1 — Summary of sequencing assembly. (A) Distribution of isotig lengths in the insect group; (B) distribution of the number of reads per isotig in the insect group;(C) distribution of isotig lengths in the bacterial group; (D) distribution of the number of reads per isotig in the bacterial group; (E) distribution of isotig lengths in the nohit group; (F) distribution of the number of reads per isotig in the nohit group. (TIF) [file pone.0035181.s001.tif]

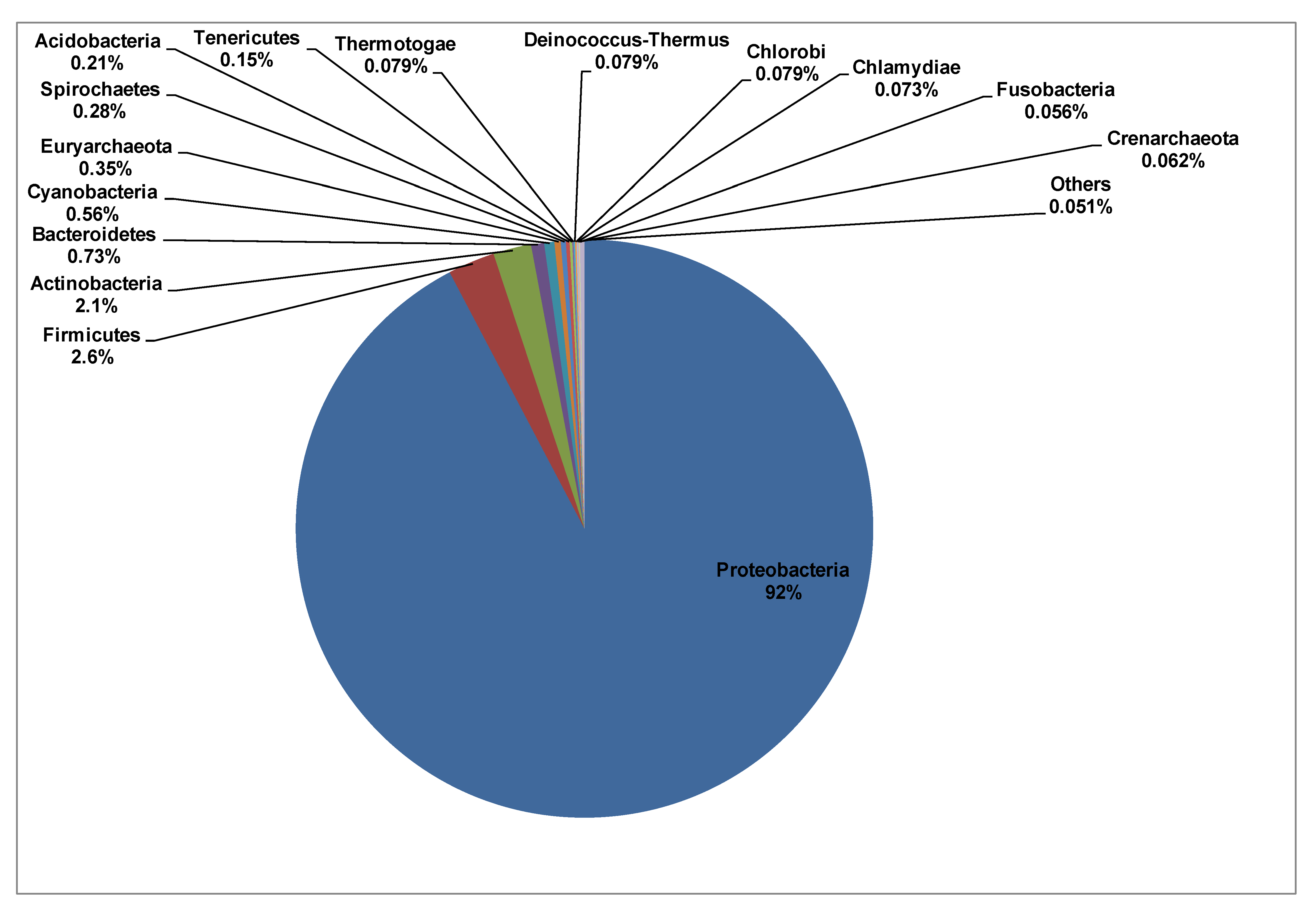

Supplement: Figure S2 — Diversity and phylogeny of bacterial symbionts in B.tabaci . Phylogenetic relationships of symbiotic bacteria in B.tabaci were resolved at the Phylum levels. (TIF) [file pone.0035181.s002.tif]

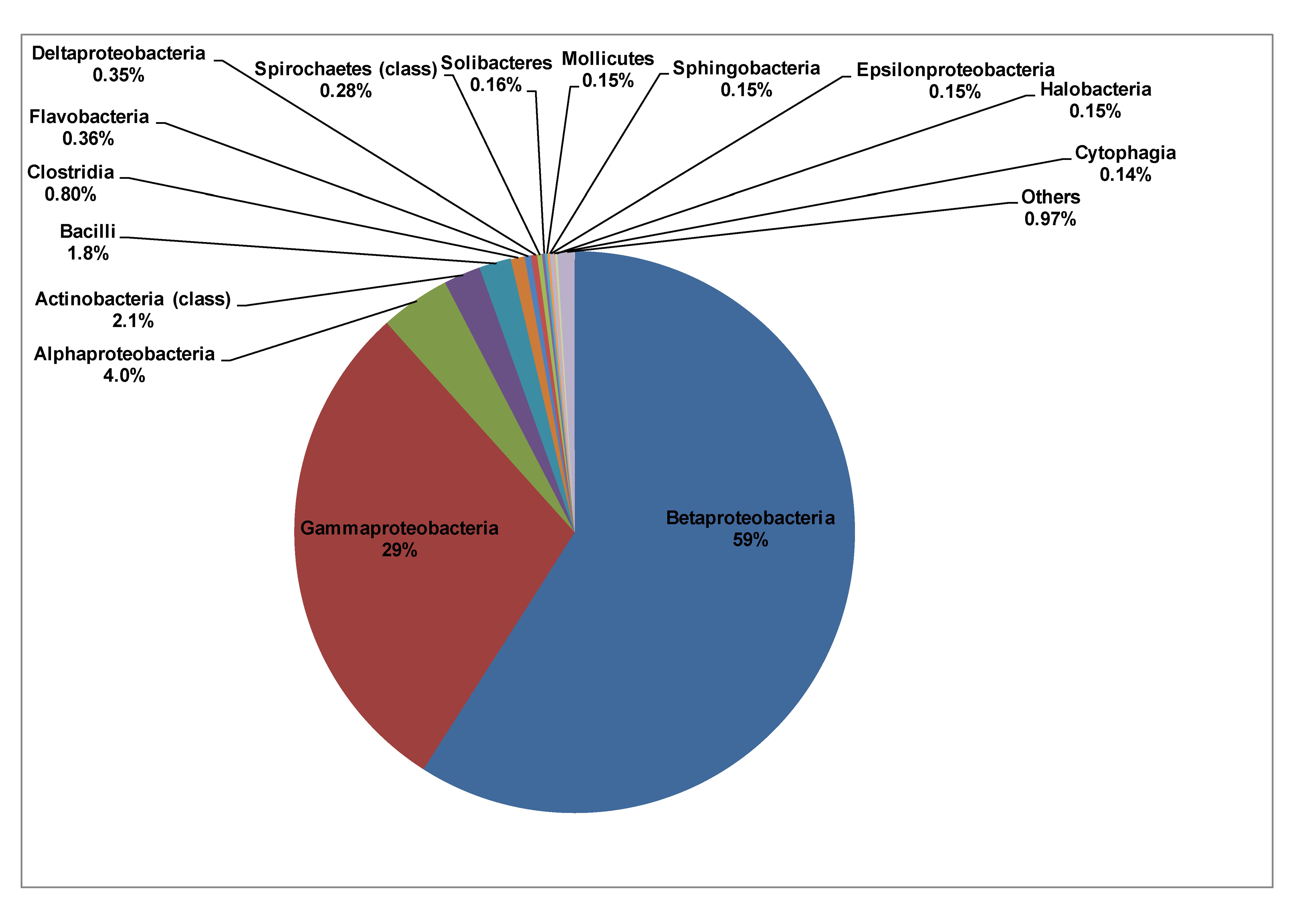

Supplement: Figure S3 — Diversity and phylogeny of bacterial symbionts in B.tabaci . Phylogenetic relationships of symbiotic bacteria in B.tabaci were resolved at the Order levels. (TIF) [file pone.0035181.s003.tif]

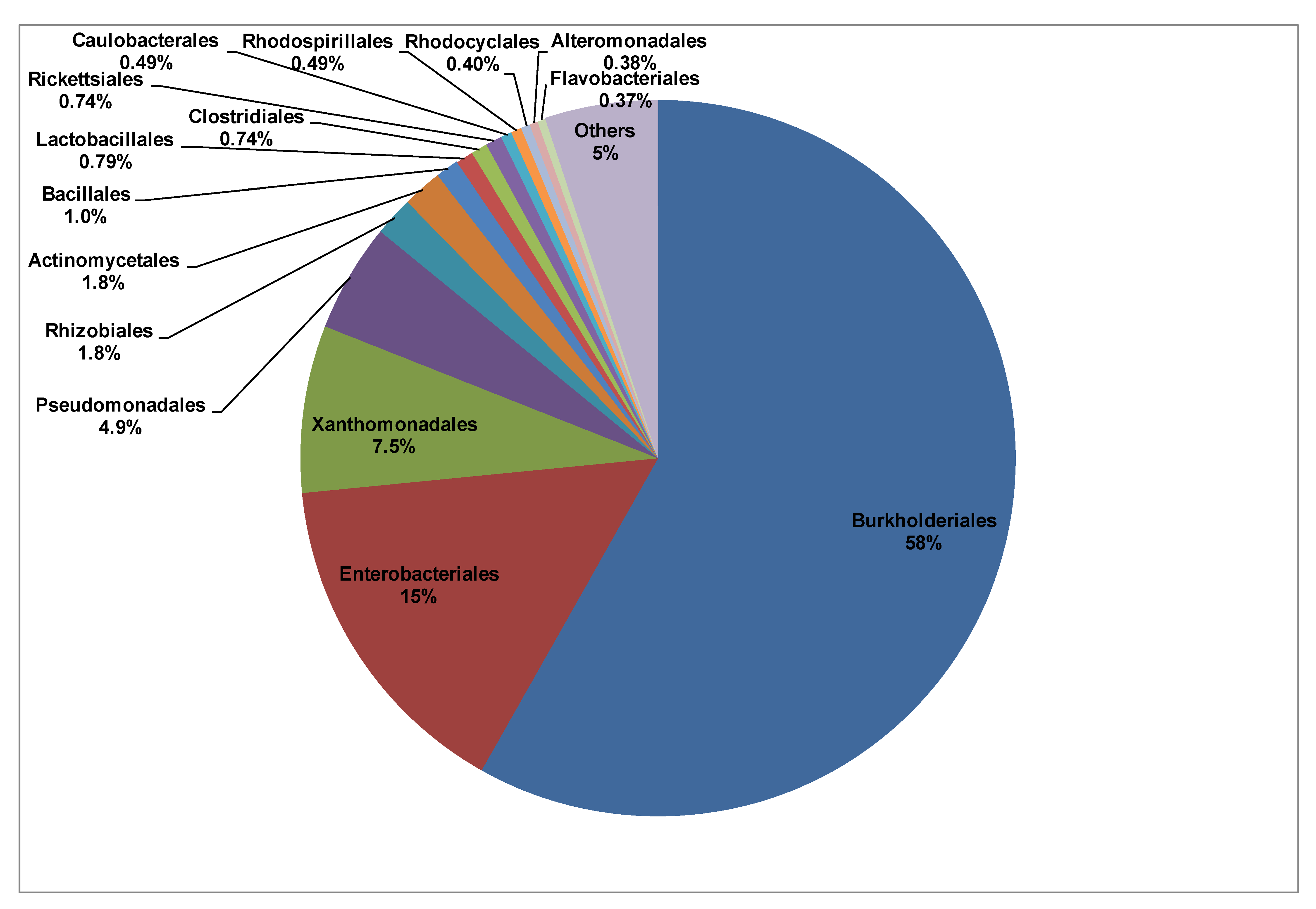

Supplement: Figure S4 — Diversity and phylogeny of bacterial symbionts in B.tabaci . Phylogenetic relationships of symbiotic bacteria in B.tabaci were resolved at the Class levels. (TIF) [file pone.0035181.s004.tif]

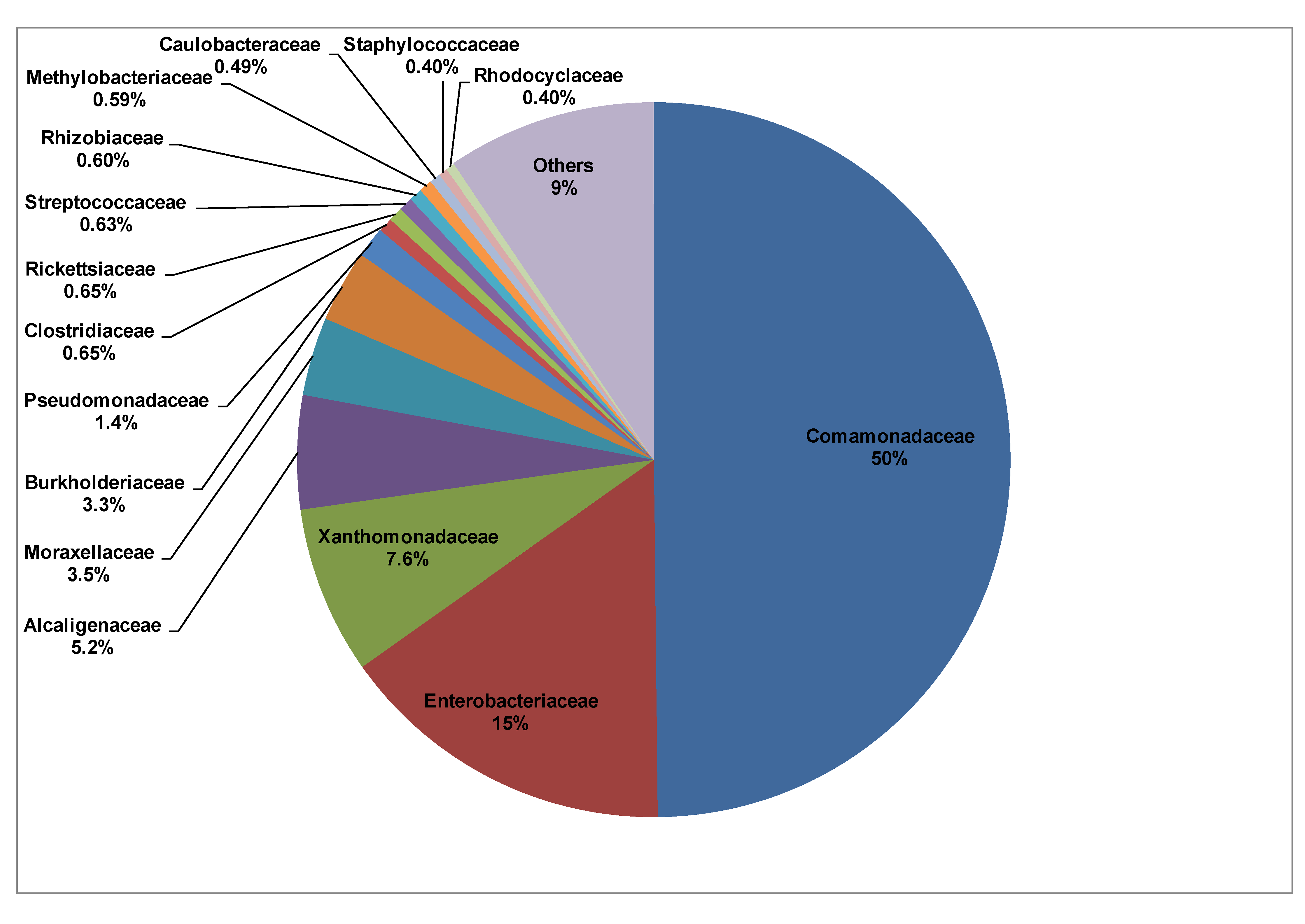

Supplement: Figure S5 — Diversity and phylogeny of bacterial symbionts in B.tabaci . Phylogenetic relationships of symbiotic bacteria in B.tabaci were resolved at the Family levels. (TIF) [file pone.0035181.s005.tif]
