# Supplementary material for: Pyrosequencing the Bemisia tabaci Transcriptome Reveals a Highly Diverse Bacterial Community and a Robust System for Insecticide Resistance
Source: PLoS One. 2012 Apr 30;7(4):e35181. doi: 10.1371/journal.pone.0035181 (PMC3340392; doi:10.1371/journal.pone.0035181)
Supplement: Table S1 — Transcriptome analyses in non-model insects using NGS platforms. (DOCX) [file pone.0035181.s006.docx]

**Table S1. Transcriptome analyses in non-model insects using NGS platforms**

| **Gene Discovery** | **Platform** | **Species (Reference)** |
| --- | --- | --- |
| SNP | 454 | *Erynnis propertius* and *Papilio zelicaon*^[24]^ |
| Tissue-specific genes expression | 454 | *Agrilus planipennis*^[36]^ *and Nilaparvata lugens*^[37]^ |
| Bt receptors | 454 | *Chrysomela tremulae*^[25]^ |
| Rice stripe virus | 454 | *Laodelphax striatellus*^[26]^ |
| Cyanogenic glucosides biosynthesis | 454 | *Zygaena filipendulae*^[27]^ |
| Immune responses | 454 | *Manduca sexta*^[28]^*and Dermacentor variabilis*^[29]^ |
| Chemosensation and sex determination | 454 | *Stomoxys calcitrans*^[30]^ |
| Insecticide targets and detoxifying enzymes | 454  Illumina | *Trialeurodes vaporariorum*^[31]^*, Bemisia tabaci*^[32]^ *and Cimex lectularius*^[33]^ |
| [Developmental](app:ds:developmental) [stage](app:ds:stage)-specific genes | Illumina | *Nilaparvata lugens*^[34]^ *and Locusta migratoria*^[35]^ |
