# Supplementary material for: Pyrosequencing the Bemisia tabaci Transcriptome Reveals a Highly Diverse Bacterial Community and a Robust System for Insecticide Resistance
Source: PLoS One. 2012 Apr 30;7(4):e35181. doi: 10.1371/journal.pone.0035181 (PMC3340392; doi:10.1371/journal.pone.0035181)
Supplement: Table S8 — Putative trehalase receptor genes in B. tabaci transcriptome. (DOCX) [file pone.0035181.s013.docx]

**Table S8 Putative trehalase receptor genes in *B. tabaci* transcriptome**

| **Sequence name** | **Sequence length (bp)** | **Accession number** | **Sequence description** | **Species** | **E-value** | **Alignment length** |
| --- | --- | --- | --- | --- | --- | --- |
| 2.0_insect_GG3J73P03G6LHD | 538 | BAH28889.1 | trehalase | *Polypedilum vanderplanki* | 4.00E-39 | 72 |
| 2.0_insect_GH93ESX02EE1V3 | 408 | BAI67864.1 | trehalase | *Artemia franciscana* | 4.00E-14 | 112 |
| 2.0_insect_GG3J73P03GCXFO | 528 | ACN85420.1 | soluble trehalase | *Nilaparvata lugens* | 3.00E-16 | 95 |
| 2.0_insect_GH93ESX02EFKEH | 346 | ACN85421.1 | membrane-bound trehalase | *Nilaparvata lugens* | 3.00E-20 | 59 |
| 2.0_insect_GH93ESX03FV33S | 402 | ACV20872.1 | membrane-bound trehalase | *Nilaparvata lugens* | 7.00E-47 | 61 |
| 2.0_insect_GH93ESX01AMDT6 | 437 | NP_001075759.1 | trehalase precursor | *Oryctolagus cuniculus* | 5.00E-08 | 92 |
| 2.0_insect_GH93ESX02EMMR4 | 163 | NP_001106141.1 | trehalase precursor | *Apis mellifera* | 2.00E-10 | 54 |
| 2.0_insect_GG3J73P03HC2NQ | 204 | XP_001602179.1 | trehalase-2 | *Nasonia vitripennis* | 5.00E-13 | 67 |
| 2.0_insect_GG3J73P03G2HTL | 509 | XP_001943494.1 | trehalase | *Acyrthosiphon pisum* | 6.00E-38 | 97 |
| 2.0_insect_isotig02448 | 1092 | XP_001949459.1 | trehalase | *Acyrthosiphon pisum* | 1.00E-122 | 269 |
| 2.0_insect_isotig02618 | 1008 | XP_001950264.1 | trehalase | *Acyrthosiphon pisum* | 2.00E-69 | 200 |
| 2.0_insect_GH93ESX01BMXCP | 267 | XP_001952111.1 | trehalase | *Acyrthosiphon pisum* | 6.00E-30 | 81 |
| 2.0_insect_isotig05746 | 501 | XP_001952606.1 | trehalase | *Acyrthosiphon pisum* | 1.00E-39 | 148 |
| 2.0_insect_GH93ESX01BYNDD | 385 | XP_393963.2 | alpha-trehalase | *Apis mellifera* | 1.00E-29 | 93 |
