# Supplementary material for: Pyrosequencing the Bemisia tabaci Transcriptome Reveals a Highly Diverse Bacterial Community and a Robust System for Insecticide Resistance
Source: PLoS One. 2012 Apr 30;7(4):e35181. doi: 10.1371/journal.pone.0035181 (PMC3340392; doi:10.1371/journal.pone.0035181)
Supplement: Table S9 — Genes putatively involved in insecticide resistance in whitefly transcriptomes. (DOCX) [file pone.0035181.s014.docx]

**Table S9. Genes putatively involved in insecticide resistance in whitefly transcriptomes**

| Gene Annotation | *Bemisia tabaci* | *Bemisia tabaci* | *Bemisia tabaci* | *Trialeurodes vaporariorum* | *Bemisia tabaci* |
| --- | --- | --- | --- | --- | --- |
| Biotype (reference)  Platform | B[39]  Sanger | B[38]  Illumina | Q[32]  Illumina | N/A [31]  454 | B [this study]  454 |
| Cytochrome P450 monooxygenase | 4 | 66 | 109 | 69 | 97 |
| Carboxylesterase | 0 | 7 | 13 | 13 | 17 |
| Glutathione S-transferase | 2 | 14 | 13 | 11 | 24 |
| Acetylcholinesterase | 0 | 0 | 3 | 2 | 4 |
| Nicotinic acetylcholine Receptor | 1 | 7 | 13 | 13 | 8 |
| GABA receptor | 0 | 2 | 4 | 1 | 3 |
| Sodium channel | 0 | 4 | 13 | 3 | 1 |
| Chloride channel | 0 | 5 | 20 | 11 | 14 |
| NADH dehydrogenase | 10 | 15 | 24 | 26 | 72 |
| NADH oxidoreductase | 2 | 10 | 7 | 17 | 6 |
| ABC transporter | 3 | 12 | 22 | 12 | 221 |
